# Health Status and Self-Perception of Health in Czech Roma Community

**DOI:** 10.1093/eurpub/ckac130.176

**Published:** 2022-10-25

**Authors:** E Volevach, M Ely, H Maršálková, L Gulová, R Mikulik

**Affiliations:** 1 The International Clinical Research Center, St. Anne's University Hospital Brno, Brno, Czechia; 2 Faculty of Medicine, Masaryk University, Brno, Czechia; 3 Faculty of Education, Masaryk University, Brno, Czechia; 4 Faculty of Humanities, Tomas Bata University, Zlin, Czechia

## Abstract

**Background:**

The Roma population is reported to have a lower life expectancy in several European countries. The reasons for this are not well described, which limits the development of effective health promotion programs. This report investigates some possible reasons: self-perception of health risks and discrimination in a sample of Czech Roma. The study is a part of a complex health awareness program.

**Methods:**

This is a pilot descriptive, cross-sectional survey conducted in Brno, Czech Republic in March 2022. Respondents were identified by community gatekeepers using quotas of gender and education. Data on disease incidence, lifestyle, attitudes to health care and risk perception were collected.

**Results:**

In the sample of 30 participants, 60% were female, average age 42±5, 60% primary education. 57% daily smokers, with an average 17 cigarettes daily during 18 years. 35% had 1 chronic disease, another 38% had 2 and more. The most prevalent diseases were hypertension (43%) and obesity (41%). 60% of respondents with hypertension as compared to 23% without hypertension considered their stroke risk as medium. 43% smokers as compared to 21% non-smokers consider their risk of COPD as medium (for lung cancer 41% vs. 0%). Roma report not visiting a doctor due to a lack of time (33%), expecting the problem to resolve itself (33%), long waiting time (33%), but not discrimination (0%).

**Conclusions:**

The Roma with risk factors correctly identify their risk of stroke, COPD, lung cancer as higher. None of them feel discriminated from physicians, but they limit contact with them due to other reasons. It appears that the prevalence of risk factors is higher in the Roma when compared to Caucasians based on the European Health Interview Survey (16% female, 31% male hypertension; 14% female, 29% male obesity). Self-perception of health and feeling of discrimination in Roma communities should be taken into account when designing health interventions.

**Key messages:**

- Roma people evaluate their risk of diseases adequately to their health status.
- The reasons for not using healthcare are not connected to discrimination.

